# Effects of motivational interviewing intervention on psychological status, compliance behavior and quality of life of patients with malignant tumor combined with diabetes mellitus

**DOI:** 10.12669/pjms.40.6.7627

**Published:** 2024-07

**Authors:** Xiao-juan Hou, Ming-yi Qin, Yue-qin Liu, Qin Zhao, Fu-jun Wang, Lei Bai

**Affiliations:** 1 Xiao-juan Hou, Department of Endocrinology, The Fourth Hospital of Hebei Medical University, Shijiangzhuang 050011, Hebei, P.R. China; 2 Ming-yi Qin, Department of Nursing, The Fourth Hospital of Hebei Medical University, Shijiangzhuang 050011, Hebei, P.R. China; 3 Yue-qin Liu, Department of Endocrinology, The Fourth Hospital of Hebei Medical University, Shijiangzhuang 050011, Hebei, P.R. China; 4 Qin Zhao, Department of Endocrinology, The Fourth Hospital of Hebei Medical University, Shijiangzhuang 050011, Hebei, P.R. China; 5 Fu-jun Wang, Department of Endocrinology, The Fourth Hospital of Hebei Medical University, Shijiangzhuang 050011, Hebei, P.R. China; 6 Lei Bai, Department of Endocrinology, The Fourth Hospital of Hebei Medical University, Shijiangzhuang 050011, Hebei, P.R. China

**Keywords:** Motivational interviewing, Malignant tumor, Diabetes mellitus, Psychological status, Compliance behavior, Quality of life

## Abstract

**Objective:**

To investigate the effects of motivational interview education on psychological status, compliance behavior and quality of life in patients with malignant tumors combined with diabetes mellitus.

**Methods:**

This is a retrospective study. Eighty patients with malignant tumors combined with diabetes mellitus admitted at The Fourth Hospital of Hebei Medical University from January 2021 to June 2022 were included as subjects and divided into observation group and control group according to the intervention measures. Patients in the control group were given routine health education intervention, while those in the observation group were given motivational interviewing intervention on the basis of the control group. We compared the prognosis, cognitive function, quality of life, relief of cancer pain before intervention and three months after the intervention of the two groups were compared.

**Results:**

At three months after the intervention, the total remission rate of cancer pain in the observation group was higher than that in the control group(p<0.05), while the levels of FBG and 2hPG in the observation group were significantly lower than those in the control group(p<0.05). Self-Rating Anxiety Scale(SAS) and Self-rating depression scale(SDS) scores decreased in both groups three months after the intervention, with the level of reduction in the observation group being higher than that in the control group(p<0.05). The overall compliance was higher in the observation group than in the control group(p<0.05).

**Conclusion:**

Motivational interviewing leads to alleviate negative emotions, improve the psychological status, enhance compliance behavior and improve quality of life in patients with malignant tumors combined with diabetes mellitus.

## INTRODUCTION

Recent years have witnessed a spurt of progress in social and economic development, improvement of medical and health standards, and earth-shaking changes in people’s lives, with the morbidity and mortality rates of once widespread parasitic and infectious diseases declining dramatically. However, non-communicable diseases are now seriously making inroads into human health^1^,^2^, among which malignant tumors and type-2 diabetes mellitus(T2DM) and their comorbidities have become a serious threat to the lives of the Chinese people.^3^ Malignant tumors are mainly triggered by the malignant proliferation of cells, manifesting as persistent, invasive, metastable local masses in the body, which will cause damage to normal tissue structures. T2DM, as a common clinical chronic disease, has been shown by epidemiological surveys^4^,^5^ to have attacked more than 425 million patients worldwide, of which the incidence of T2DM in China is about 10.5%, ranking first among major diseases.

As a result, chronic lesions and functional decline occur in tissues and organs such as blood vessels, hearts and nerves, posing a serious threat to the health and life safety of patients.^6^ A study^7^ pointed out that T2DM can induce a variety of malignant tumors, and significantly increase the risk of malignant tumors in T2DM patients, whose incidence of malignant tumors is higher than that of non-T2DM patients. For those with malignant tumors combined with T2DM, there are more complex metabolic disorders and serious diseases, most of which will have frequently recurrent diseases and poor treatment effects. Strengthening clinical interventions on patients with malignant tumors combined with T2DM is of great significance to improve the therapeutic effect of malignant tumors, stabilize the blood sugar level of patients, reduce negative emotions and improve their quality of life. Based on this, the clinical effect of the motivational interviewing intervention on patients with malignant tumors combined with chemotherapy for T2DM was analyzed in this paper.

## METHODS

This is a retrospective study. Eighty patients with malignant tumors combined with T2DM admitted at The Fourth Hospital of Hebei Medical University from January 2021 to June 2022 were included as subjects and divided into observation group and control group according to the random number method with 40 cases in each group.

### Ethical Approval

The study was approved by the Institutional Ethics Committee of The Fourth Hospital of Hebei Medical University (No.:2023KS118; date:February 05, 2023), and written informed consent was obtained from all participants.

### Inclusion criteria:

- Patients meeting the diagnostic criteria for malignant tumor and T2DM.
- Patients ≥18 years old.
- Patients with stable medical conditions.
- Patients without cognitive impairment and with normal communication and text reading ability.
- Patients receiving antitumor therapy with chemical drugs.
- Patients who gave informed consent to the study and signed the informed consent form.

### Exclusion criteria:

- Patients with psychiatric disorders and unable to communicate normally.
- Patients with combined vital organ insufficiency.
- Patients with poor compliance and who did not cooperate with the study.
- Patients with incomplete clinical data and lost to follow-up.

All patients were treated immediately after admission. Patients in the control group were given conventional health education interventions. After admission, patients’ general information and psychological status were collected, treatment files were established, and centralized lectures and education were given to patients. The physicians and nurses actively communicated with patients to understand their inner feelings and current troubles, and also talked to the patients’ families about the relevant contents of malignant tumor and diabetes treatment and daily care, emphasizing the importance of ensuring patients’ psychological guidance, compliance behaviors and quality of life.

Patients in the observation group were given motivational interviewing intervention on the basis of the control group. A one-on-one interview was conducted every three weeks for 20-30 sminutes each time three times in total. During the first interview, appropriate individualized communication was chosen according to the patients’ current specific conditions, so as to figure out the patients’ daily living habits and disease situation. In this way, the problems existing in patients’ disease response and their ambivalence in the treatment process were preliminarily clarified, and the advantages and disadvantages of patients’ good psychological status and compliance behavior as well as the importance of improvement were explained in a targeted manner. All the questionnaire was given to the participants for investigation, and then collected in about five minutes.

During the second interview, the gap between the patients’ main ambivalence and the desired goal was further reinforced, and patients were encouraged to speak out their inner troubles, potential conflicts and ways to solve problems, etc., so as to explain the treatment and prognosis of the disease concerned by patients and their families to alleviate patients’ worries, guided them to understand the risks and benefits of compliance with daily living arrangements, taking analgesic drugs and controlling blood sugar for the disease, assisted them in developing plan in improve their own behaviors, and ultimately, developed a rehabilitation program.

During the third interview, patients were recognized for their progress and given appropriate incentives to enhance their confidence in adhering to their behaviors. Meanwhile, family members were encouraged to continue to urge the patients to maintain good behavior and compliance behavior. Targeted guidance programs were provided to help them find the final solution according to their own conditions. Long-term contacts were established with patients so that they could receive help whenever they needed advice or guidance. Mean follow up period was six months.

### Observation indexes:

Comparison of cancer pain remission between two groups at three months after the intervention: complete remission: the disappearance of pain after the intervention without recurrence; partial remission: significant reduction of pain after the intervention, which basically did not disturb sleep and allowed the patients to carry out normal daily life; mild remission: a certain degree of pain remission after the intervention, but the pain was still more obvious and seriously disturbed sleep; no remission: no improvement or even worsening of the pain level compared to before the intervention. Total remission rate =

### Blood glucose level

Fasting blood glucose (FBG) in the morning and two hours postprandial blood glucose(2hPG) levels of the two groups before and three months after the intervention were compared and tested by a Johnson&Johnson blood glucose tester and the matching blood glucose test strips.

### Psychological status

Self-rating anxiety scale (SAS) and self-rating depression scale (SDS) were employed to compare the anxiety and depression of the two groups before and three months after the intervention. Both scales covered 20 questions each, with a total score of 20-80. Likert Level four Scale was used in both scales, in which anxiety was considered if the SAS score was above 50, and depression was considered if the SDS score was above 53.

### Compliance behavior

The differences in compliance behavior between the two groups after the intervention were compared. *Full compliance:* taking medication in strict compliance with medical advice, regular work and rest, reasonable diet and being able to review regularly, and basically mastering knowledge related to the disease. Partial compliance: taking the medication regularly and quantitatively under supervision, having difficulty in complying with one or two of the items of health knowledge, health behavior and regular review. Non-compliance: not taking medication in compliance with medical advice, irregular diet and rest, poor knowledge of health, and irregular review. Overall compliance rate = (complete compliance + partial compliance)/total number of cases × 100%.

### Quality of life

quality of life and self-care assessment: The Stroke-Specific Quality-of-Life (SSQOL) was used to assess the patients’ quality of life in four aspects: language, role, cognition and somatic function, respectively, before and after the intervention. Each item was scored on a scale of 0 to 20, with higher scores indicating better quality of life.

### Statistical analysis

All data in this study were statistically analyzed by SPSS 21.0 software, and measurement data were expressed as mean ± standard deviation (*χ̅*±*S*). Two independent sample t-test was used for comparison between groups, paired t-test was used to analyze data within groups. Qualitative data were represented by the number of cases and percentage (%), and c^2^ test was performed for comparison between groups. *P<*0.05 indicates a statistically significant difference.

## RESULTS

No significant difference was observed in the comparison of general data between the two groups, which was comparable (Table-I). The overall remission rate of cancer pain in the observation group was higher than that in the control group, with a statistically significant difference (*p*<0.05) (Table-II).

**Table-I:** Comparative analysis of general data between the observation group and the control group.

Item	Observation group (n=40)	Control group (n=40)	t/c^2^	P
Age (years)	56.48±10.66	56.08±8.98	0.182	0.856
Gender (Male/Female)	25/15	23/17	0.208	0.648
Duration of malignant tumor (years)	4.23±0.73	4.10±0.84	0.708	0.481
Duration of diabetes mellitus (years)	6.55±1.58	6.85±1.75	0.804	0.424
Tumor type			1.988	0.851
Breast cancer	5 (12.50%)	4 (10.00%)		
Stomach cancer	7 (17.50%)	7 (17.50%)		
Lung cancer	6 (15.00%)	9 (22.50%)		
Colon cancer	6 (15.00%)	7 (17.50%)		
Thyroid cancer	3 (7.50%)	1 (2.50%)		
Esophageal cancer	3 (7.50%)	2 (5.00%)		

**Table-II:** Comparison of cancer pain remission between the two groups after the intervention [Cases (%)].

Group	Complete remission	Partial remission	Mild remission	No remission	Overall remission rate
Observation group (n=40)	1 (2.50)	21 (52.50)	15 (37.50)	3 (7.50)	37 (92.50)
Control group (n=40)	0 (0.00)	16 (40.00)	12 (30.00)	12 (30.00)	28 (70.00)
c^2^ value					6.646
P value					0.010

No significant difference was observed in the FBG and 2hPG levels of the two groups before the intervention(*p*>0.05), and the FBG and 2hPG levels of the observation group were significantly lower than those of the control group three months after the intervention, with a statistically significant difference(*p*<0.05) (Table-III). No significant difference was observed in the SAS and SDS scores of the two groups before the intervention(*p*>0.05). The SAS and SDS scores of both groups decreased three months after the intervention, and the level of decrease was higher in the observation group than in the control group, with statistically significant differences(*p*<0.05) (Table-IV).

**Table-III:** Comparison of blood glucose levels between the two groups before and after the intervention (*χ̅*±*S*).

Group	FBG		2hPG	

	Before intervention	After intervention	Before intervention	After intervention
Observation group (n=40)	9.60±3.47	5.92±2.16	13.69±3.73	8.09±1.958
Control group (n=40)	9.44±3.19	7.02±2.18	14.37±2.62	9.54±2.10
t value	0.210	2.266	0.942	3.201
P value	0.835	0.026	0.349	0.002

**Table-IV:** Comparison of SAS and SDS scores between the two groups before and after the intervention (*χ̅*±*S*).

Group	SAS		SDS	

	Before intervention	After intervention	Before intervention	After intervention
Observation group (n=40)	57.23±1.42	35.80±2.38	56.93±1.38	36.05±2.82
Control group (n=40)	57.60±1.58	40.93±2.06	57.33±1.44	42.80±2.38
t value	1.115	10.314	1.267	11.577
P value	0.268	0.000	0.209	0.000

The overall compliance rate in the observation group was higher than that in the control group, with a statistically significant difference(*p*<0.05) (Table-V). No statistically significant difference was observed between the two groups in the SS-QOL scores before the intervention(*p*>0.05). Three months after the intervention, the SS-QOL scores of both groups was significantly improved compared with those before the intervention, and the improvement degree of the observation group was significantly better than the control group, with statistically significant differences(*p*<0.05) (Table-VI).

**Table-V:** Comparison of compliance behaviors between the two groups [Cases (%)].

Group	Full compliance	Partial compliance	No compliance	Overall compliance rate
Observation group (n=40)	23 (57.50)	12 (30.00)	5 (12.50)	35 (87.50)
Control group (n=40)	16 (40.00)	11 (27.50)	13 (32.50)	27 (67.50)
c^2^ value				4.588
P value				0.032

**Table-VI:** Comparison of SS-QOL and ADL scores between the two groups before and after the intervention (*χ̅*±*S*).

Group	Somatic function		Language function		Role function		Cognitive function	

	Before intervention	After intervention	Before intervention	After intervention	Before intervention	After intervention	Before intervention	After intervention
Observation group	4.78±0.42	10.93±0.89	5.93±0.69	15.00±1.09	5.70±0.52	11.55±0.90	7.75±0.54	18.40±0.98
Control group	4.60±0.71	7.53±0.96	5.60± 0.90	11.60±1.22	5.60±0.67	11.43±1.06	7.63±0.67	14.40±1.15
t value	1.341	16.437	1.809	13.194	0.746	18.729	0.919	16.728
P value	0.184	0.000	0.074	0.000	0.458	0.000	0.361	0.000

## DISCUSSION

In this paper, the FBG and 2hPG levels of patients in the observation group after motivational interviewing intervention were significantly lower than those in the control group, and the remission rate of cancer pain in the observation group was lower than that in the control group(*p*<0.05), indicating the role of motivational interviewing intervention in improving patients’ conditions and enhancing treatment effects. Malignant tumors are a major public health problem that seriously affects human life and health, ranking first in the mortality rate of urban residents for a long time.^8^,^9^ Relevant studies have shown that^10^ T2DM increases the risk of malignant tumors and is a vital risk factor for the formation of various malignant tumors. Patients with malignant tumors combined with T2DM often suffer from a variety of psychological problems, which in turn have an impact on their quality of life.

The concept of “health” proposed by WHO emphasizes the multi-dimension of health and the wholeness of life. Malignant tumors and T2DM, as chronic diseases, each have an impact on patients’ psychological status and quality of life. If the two are combined, the impact on patients will be superimposed, causing the deterioration of their condition. Modern medicine is committed to prolonging the survival period of patients with malignant tumors while enabling good maintenance of patients’ quality of life. To this end, strengthening clinical interventions on patients with malignant tumors combined with T2DM is of great significance to improve treatment outcomes, prolong life and ameliorate the quality of life of patients.^11^,^12^ Treating patients with malignant tumors combined with T2DM is at increased difficulty compared to treating either one of the two alone. It is important to focus not only on chemotherapy interventions during chemotherapy for malignant tumors, but also to actively control patients’ blood glucose levels to avoid or reduce the occurrence of severe complications.

Clinically, conventional interventions on patients with malignant tumors combined with T2DM are patient-centered with emphasis on the improvement of patients’ physical conditions. However, the large differences among individual patients lead to a certain passivity and blindness of conventional interventions^13^, which, together with the lack of targeted interventions on patients’ psychological states, make the interventions have limited effects. Motivational interviewing, originally applied by Miller, a professor of psychology and psychiatry in the United States^14^, is now widely used in medication control, patient compliance behavior, chronic disease treatment and behavior change, etc. It is a collaborative and goal-oriented style of communication that places emphasis on the adjustment of patients’ psychological state.

During the interview, patients’ concerns are answered so as to demonstrate good expectations and increase their confidence in the future, and patients are invited to explore new possibilities and solutions to problems by assessing at which stage of behavior change the intervention subject is in, and then strategic approaches are applied to facilitate the behavior change of the intervention subject. It was shown in a study by Hardcastle et al.^15^ that one-on-one motivational interviewing for the elderly with chronic diseases can effectively improve the overall level of patients’ psychological status and medication compliance. It has also been shown^16^ that multiple interviews are more effective than a single long interview. Afterward, another study showed^17^ that the adoption of motivational interviewing for medication compliance in HIV patients resulted in a shorter intervention effect and better outcome than the traditional intervention model, with a significant increase in patient autonomy and motivation to participate.

It was shown in this study that the SAS and SDS scores in the observation group were lower than those in the control group after the adoption of motivational interviewing, and the overall compliance rate in the observation group was better than that in the control group(*p*<0.05), which was consistent with the literature. Long-term disease suffering due to malignant tumors, the timing of treatment, and patient psychological status directly affect the quality of life of patients. Studies have shown that the poorer the psychological status of patients with malignant tumors combined with T2DM, the lower the quality of life.^18^ By emphasizing the importance of self-efficacy and positive change, motivational interviewing encourages patients to change undesirable behaviors, aids patients in focusing on specific, achievable behavioral goals and improves patients’ compliance behaviors.

It was shown in this study that the language, role, cognition and somatic function of SS-QOL scores of quality of life after motivational interviewing intervention were higher than those in the control group(*p*<0.05), which was consistent with the results of relevant studies, indicating the role of motivational interviewing in improving patients’ self-management and enhancing their quality of life.

### Limitations of the study

Fewer observation cases were included, and the follow-up time was short. More patients will be included in the follow-up study, the follow-up time will be extended, so as to more objectively evaluate the advantages and disadvantages of motivational interviewing boasts and benefit more patients.

## CONCLUSIONS

Motivational interviewing boasts various benefits in treating patients with malignant tumors combined with diabetes mellitus, such as significantly improving patients’ psychological status, encouraging patients to face the disease and treatment with a positive and optimistic attitude, increasing patients’ compliance behavior, and ameliorating patients’ quality of life.

### Authors’ Contributions:

**XH** and **MQ:** Carried out the studies, data collection, drafted the manuscript, are responsible and accountable for the accuracy or integrity of the work.

**YL:** and **QZ:** Performed the statistical analysis and participated in its design.

**FW** and **LB:** Participated in acquisition, analysis, or interpretation of data and drafting of the manuscript.

All authors read and approved the final manuscript.
